# Potential effect of prior raccoonpox virus infection in raccoons on vaccinia-based rabies immunization

**DOI:** 10.1186/1471-2172-9-57

**Published:** 2008-10-03

**Authors:** J Jeffrey Root, Robert G McLean, Dennis Slate, Kathleen A MacCarthy, Jorge E Osorio

**Affiliations:** 1 United States Department of Agriculture, Wildlife Services, National Wildlife Research Center, Fort Collins, CO 80521, USA; 2 United States Department of Agriculture, Wildlife Services, National Rabies Management Program, Concord, NH 03301, USA; 3 University of Wisconsin – Madison, School of Veterinary Medicine, Madison, WI 53706, USA

## Abstract

**Background:**

The USDA, Wildlife Services cooperative oral rabies vaccination (ORV) program uses a live vaccinia virus-vectored (genus *Orthopoxvirus*) vaccine, Raboral V-RG^® ^(V-RG), to vaccinate specific wildlife species against rabies virus in several regions of the U.S. Several naturally occurring orthopoxviruses have been found in North America, including one isolated from asymptomatic raccoons (*Procyon lotor*). The effect of naturally occurring antibodies to orthopoxviruses on successful V-RG vaccination in raccoons is the focus of this study.

**Results:**

Overall, raccoons pre-immunized (n = 10) with a recombinant raccoonpox virus vaccine (RCN-F1) responded to vaccination with V-RG with lower rabies virus neutralizing antibody (VNA) titers than those which were not pre-immunized (n = 10) and some failed to seroconvert for rabies VNA to detectable levels.

**Conclusion:**

These results suggest that the success of some ORV campaigns may be hindered where raccoonpox virus or possibly other orthopoxvirus antibodies are common in wildlife species targeted for ORV. If these areas are identified, different vaccination strategies may be warranted.

## Background

Oral rabies vaccination (ORV) programs for raccoons are common in the eastern U.S. Raboral V-RG^® ^(V-RG) is the only licensed oral rabies vaccine for wildlife in the U.S. This vaccine utilizes a live vaccinia virus (genus *Orthopoxvirus*) vector. Because oral rabies vaccination rates in raccoons (*Procyon lotor*) can be low in some areas [1], and large expenses are involved in these types of campaigns [2], it is important that all key factors that could affect vaccination rates be addressed.

Several naturally occurring orthopoxviruses can be found throughout the world. For example, bank voles (*Clethrionomys glareolus*), woodmice (i.e., long-tailed field mice: *Apodemus sylvaticus*) and Nowray lemmings (*Lemmus lemmus*) yielded evidence of orthopoxvirus antibodies in Norway [3]. Similar associations were noted for select carnivore species in Fennoscandia [4] and red fox (*Vulpes vulpes*) in Germany [5]. In the U.S., endemic orthopoxvirus examples include volepox virus found in California voles (*Microtus californicus*; [6]), raccoonpox virus found in raccoons [7,8], and skunkpox virus found in a striped skunk (*Mephitis mephitis*; reviewed by [9]). Additional unrecognized orthopoxviruses may exist in the U.S. For raccoonpox and skunkpox viruses, it is unknown whether the species from which these viruses were obtained are the true reservoir species (e.g., these viruses, like many other orthopoxviruses, may be rodent-hosted), as little field work has been conducted on these viruses since their discovery.

It is unknown whether previous exposure to these North American orthopoxviruses can inhibit the effectiveness of V-RG vaccination in raccoons. However, it has been noted that preexisting immunity to vaccinia virus may impede the replication of vaccinia virus vectors and decrease the response to the recombinant product [10]. The subject of preexisting immunity has received some attention for select vectored vaccines. For example, researchers [11] indicated that mucosal vaccination overcame the barrier of preexisting orthopoxvirus immunity of mice initially immunized subcutaneously. Thirty-day old red fox (*Vulpes vulpes*) cubs with maternally derived antibodies from V-RG serologically responded (i.e., rabies virus neutralizing antibody {VNA}) to oral vaccination with V-RG [12]. However, mice primed with avirulent ectromelia virus survived a challenge with virulent ectromelia virus (genus *Orthopoxvirus*) with no signs of morbidity [13]. In mice pre-immunized with vaccinia virus and subsequently vaccinated with a recombinant vaccinia virus, antibody titers against the recombinant gene product were lower and lasted for a shorter duration [14]. In contrast, similar studies of adenoviruses suggested that the efficacy of oral vaccination is relatively unaltered by preexisting neutralizing antibodies to the vaccine carrier [15]. For vaccinia virus, the mechanism for such compromised immunity is thought to be associated with long-term anti-vaccinia immunological memory, which inhibits the replication/priming of the second vaccinia recombinant virus such that the amount of the non-vaccinia antigen produced by the recombinant vector is no longer sufficient [14,16].

The effects of pre-existing immunity on vectored vaccines have not been rigorously studied and different conclusions have been drawn. Because this relationship has not been thoroughly addressed for the oral vaccination of raccoons, the objective of this study was to determine whether exposure to raccoonpox virus inhibits the successful V-RG vaccination and development of rabies VNA titers in raccoons in an experimental pen setting.

## Results

### Orthopoxvirus antibodies

Pre-treatment serology indicated that seven (four control and three treatment) of twenty raccoons may have been exposed to an orthopoxvirus prior to their capture, albeit their antibody titers (1:5) were all at the threshold of detection (geometric mean titer {GMT} < 1:5; treating all < 1:5 titers as one significant digit less than the detection limit). Assays on a second pre-treatment blood sample were identical with one exception; the titer of one raccoon increased from < 1:5 (e.g., below the detection limit) to 1:5 during its quarantine period prior to experimentation (GMT < 1:5). Of these animals with pre-existing orthopoxvirus antibodies at low titers, all yielded evidence of rabies VNA following their second dose of V-RG.

Thirty days after a single dose (DPI) with the RCN-F1 vaccine (10^8 ^pfu), nine of 10 RCN-F1 treated raccoons (treatment) had detectable antibodies to raccoonpox virus (i.e., ≥1:5; range = < 1:5 – 1:20; GMT = 1:7). At that time, seven of 10 control raccoons (those only vaccinated with V-RG) were negative for antibodies reactive with this test; the remaining three animals had pretreatment antibodies (range < 1:5 – 1:5; GMT = < 1:5). Sixty-nine days following the single RCN-F1 vaccination (36 days after the first V-RG vaccination {10^7 ^pfu}), nine of 10 treatment animals had raccoonpox virus antibodies (range < 1:5 – 1:20; GMT = 1:12), while three of 10 control animals had sera that was reactive with this test (likely limited cross-reactivity of antibodies to the vaccinia virus in V-RG in some animals; range < 1:5 – 1:15; GMT = < 1:5). One animal was consistent with its background levels, but the titer of the other two animals increased. Ninety-seven DPI of the RCN-F1 vaccination, all treatment animals had positive raccoonpox virus antibody titers (range 1:5 – 1:15; GMT = 1:13), while the sera of four of 10 control raccoons were positive in this assay (range < 1:5 – 1:15; GMT = 1:6). The final detection for raccoonpox virus antibodies was performed on serum samples from 149 DPI of RCN-F1 vaccination (31 days following the second dose of V-RG {10^8 ^pfu}). Of the serum samples collected from that time point, all treatment samples were raccoonpox virus antibody positive and had identical titers to those samples collected at 97 DPI (range 1:5 – 1:15; GMT = 1:13), while six of 10 control samples had antibodies reactive with the test (range < 1:5 – 1:15; GMT = 1:7).

### Rabies virus neutralizing antibodies

**Figure 1:**
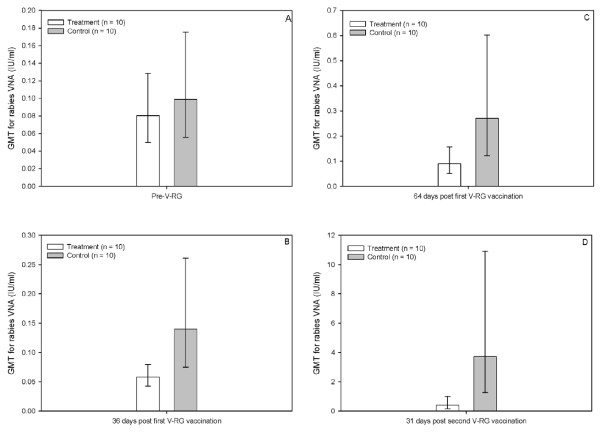
**Graphical representation of rabies virus neutralizing antibodies (VNA) in raccoons during four time points including pre-experiment** (A), 36 days post first V-RG vaccination (B), 64 days post first V-RG vaccination (C), and 31 days post second V-RG vaccination. Treatment animals are those raccoons pre-vaccinated with RCN-F1 (a raccoonpox virus vectored plague vaccine, see reference 25), while control animals are those which were not vaccinated with RCN-F1. The second V-RG vaccination was administered 85 days after the first vaccination (see discussion).

Seven of the raccoons that were used in this experiment had evidence of pre-treatment/natural rabies VNA (four control and three treatment raccoons). The positive antibody titers ranged from 0.20 – 0.80 IU/ml at that time. The titers of six of the seven raccoons (three of four control animals and all treatment animals) decreased in a subsequent serum sample. Prior to treatments being employed, the geometric mean titers (treating all < 0.05 IU/ml titers as one significant digit less than the detection limit) were 0.10 and 0.08 IU/ml for the control and treatment groups, respectively (Figure 1A).

Thirty-six days after the first V-RG vaccination (e.g., 10^7 ^pfu), seven of 20 raccoons yielded rabies VNA at detectable levels (≥ 0.05 IU/ml; range < 0.05 – 0.79 IU/ml; Figure 1B). Six of the seven were those with the pre-existing rabies VNA mentioned above. One treatment raccoon had detectable rabies VNA, albeit it was likely the pre-existing antibodies that were detected, as its titer decreased following vaccination. The GMTs were 0.14 and 0.06 IU/ml for the control and treatment groups, respectively. Sixty-four days after this vaccination, 10 of 20 raccoons yielded rabies VNA at detectable levels (titer range < 0.05 – 1.6 IU/ml; Figure 1C). Three of 10 treatment animals had detectable rabies VNA at that time point (range < 0.05 – 0.40 IU/ml). The GMTs were 0.27 and 0.09 IU/ml for the control and treatment groups, respectively.

Thirty-one days following the second V-RG vaccination (10^8 ^pfu), 17 of 20 raccoons yielded evidence of rabies VNA. The three that failed to seroconvert to detectable levels were treatment animals. The rabies VNA titer range for the control group was 0.20 to 67.0 IU/ml (7 of 10 animals ≥ 1.0 IU/ml), while the titer range for the treatment group was < 0.05 – 2.6 IU/ml (3 of 10 animals ≥ 1.0 IU/ml). The GMTs at this time point were 3.72 and 0.40 for the control and treatment groups, respectively (Figure 1D). The ratio of GMTs for rabies VNA from control to treatment raccoons was 9.3.

### Histological findings

Following post-mortem examinations (day 252), gross and histologic findings were consistent with previous reports of naturally occurring disease in wild raccoons. The most commonly noted findings included chronic interstitial nephritis, periportal hepatitis, myocardial sarcocysts and pneumoconiosis. Two raccoons had mild, chronic inflammation in the choroid plexus and a single animal had a hepatic adenoma. The lack of pathology that can be definitively linked to the two orthopoxvirus-vectored vaccines deployed further supports the safety of both of these vaccines, even after three doses.

## Discussion

Although the raccoons used in this study were captured in Colorado, an area free of enzootic raccoon rabies, seven of the raccoons that were used in this experiment had evidence of pre-treatment rabies VNA. Thus, it is possible that these animals were exposed to a different rabies virus variant (e.g., bat), as this has been suggested as the cause of rabies VNA in striped skunks (*Mephitis mephitis*) sampled in areas thought to be free of skunk rabies in Alberta [17]. It is also possible that they may have been previously captured, vaccinated, and released, a common practice among wildlife rehabilitators. Another, possibly the most parsimonious, reason for the observation of the pretreatment antibodies is non-specific virus inhibition. Of interest, we and others have observed RFFIT positive sera samples from raccoons collected in areas thought to be free of raccoon rabies in multiple states. Future studies should assess whether RFFIT positive samples observed in wildlife sera obtained from areas free of terrestrial rabies virus variants and ORV zones are actually positive due to specific antibodies or other, non-specific factors. Notably, Bahloul et al. presented data on presumably unvaccinated dogs with fairly high background antibody rates and observed distinct serological differences in dogs vaccinated in an experimental (pen) setting versus those in the field [18]. As such, they suggested that a cutoff threshold of 0.5 IU/ml should be interpreted cautiously and a higher threshold might be more appropriate in the evaluation of rabies immunity in natural settings to marginalize various interfering factors [18].

Six of 10 control raccoons yielded evidence of antibodies reactive with raccoonpox virus. However, some of the animals which failed to seroconvert for orthopoxvirus antibodies (i.e., vaccinia virus antibodies reacting with raccoonpox virus in the microneutralization test) to detectable levels did seroconvert for rabies virus, occasionally to high titers (e.g., > 10.0 IU/ml). Thus, only a limited antibody response to vaccinia virus may occur in raccoons when vaccinated with V-RG. However, considering that the majority of these animals did seroconvert, a more probable explanation is that the assay we used may only be marginally able to detect vaccinia virus antibodies, as this assay utilizes raccoonpox virus for neutralization.

Raccoons were dosed with V-RG twice because post-hoc plaque assays indicated that our stock vaccine was approximately one log lower than expected (e.g., 10^8 ^pfu/ml rather than 10^9 ^pfu/ml) and initial serological results indicated low vaccination rates in both groups of raccoons. Nonetheless, the GMT differences of the treatment and control groups in this study form a trend. Thirty-three days after the initial dose of V-RG (10^7 ^pfu), the treatment group yielded a GMT of 0.06 (CI = 0.0425 – 0.0795), while the control group yielded a GMT of 0.14 IU/ml (CI = 0.0751 – 0.2610; Figure 1B). Sixty-four days following this initial vaccination, the GMTs increased to 0.09 (CI = 0.0509 – 0.15693) and 0.27 IU/ml (CI = 0.1213 – 0.6018) for the treatment and control groups, respectively (Figure 1C). Thirty-one days after the second dose of V-RG (10^8 ^pfu), the GMTs increased to 0.40 IU/ml (CI = 0.1635 – 0.9921) for the treatment group and 3.72 IU/ml (CI = 1.2682 – 10.9150) for the control group (Figure 1D). Thus, the treatment we employed appears to have had an effect on the V-RG induced seroconversions and the rabies VNA titers produced in these raccoons. This is apparent when one considers that some of the pre-immunized (treatment) animals failed to seroconvert for rabies VNA to detectable levels, even after two doses of V-RG, whereas all control animals seroconverted. Nonetheless, the majority of the treatment animals did seroconvert for rabies VNA to detectable levels, albeit their responses were variable among individuals. Our observation may be consistent with others [14], who noted that the immunological memory against vaccinia virus inhibited the replication of a vaccinia recombinant during the second infection, which also resulted in a suppressed response against the recombinant gene product. Our vaccination results are also confirmed by *in vitro *studies (JEO's Laboratory) indicating that sera with anti-raccoonpox virus antibodies can cause about a 1 log reduction in vaccinia virus titers in the modified neutralization test we employed. The cross-reactivity between raccoonpox virus and other orthopoxviruses has been previously described by others. Raccoonpox virus produces a hemagglutining (HA) antigen that is inhibited by an antiserum to vaccinia virus [7]. Alexander et al. reported that sera from raccoons with antibody titers to raccoonpox virus partly cross-reacted with vaccinia virus by the hemagglutining inhibition (HAI) assay [19]. Furthermore, in an experimental study a raccoon inoculated with raccoonpox virus developed high HAI titers of 1:320 against raccoon poxvirus at 5 weeks post-inoculation [8]. The sera had also a HAI titer of 1:40 against vaccinia virus [8].

Even though there was a distinct difference in the rabies VNA GMTs between the treatment and control groups, we did note a boosting effect in most animals following their second V-RG vaccination. Thus, these data also suggest the possibility that a second high dose of V-RG might not be inhibited by previous vaccination.

Although rabies VNA titers are not widely suitable as a generic criterion for successful rabies vaccination in wildlife [20], recent studies imply an increasing proportion of raccoons are protected at higher titers [21]. For example, in a study using three groups of raccoons considered to have low-positive, medium-positive, and high-positive (> 0.45 IU/ml) rabies VNA titers, the highest level of post-challenge survivorship (83%) was obtained in the group considered high-positive; the other two groups were significantly lower [21]. Of interest, low titers of antibodies to raccoonpox virus may have completely inhibited V-RG vaccination in three treatment animals and likely reduced the rabies VNA titers in others. However, some treatment animals yielded clear vaccination responses to V-RG. Nonetheless, the low rabies VNA titers we observed in several vaccinated treatment animals suggests that raccoons with preexisting antibodies to raccoonpox virus will, on average, have lower antibody titers following V-RG vaccination. It should be noted, however, that rabies VNA are only a part of the protective immune response produced by V-RG, as other immunological processes may also be important for the quality of the immune response.

Antibodies to naturally occurring orthopoxviruses in ORV target species will likely be patchy in distribution. Old World orthopoxviruses provide a well-studied example. Boulanger and others [22] did not detect antibodies to orthopoxviruses in wild red foxes in South Belgium (n = 72). A similar association was noted in France and various parts of Belgium [23]. However, marginally high seroprevalence rates were found in red foxes in Germany (at least 16%) [5], Norway (11%), and Finland (50%; [4]). The large-scale geographic variability of the aforementioned results suggests that similar variability might be found in other locations.

## Conclusion

Orthopoxviruses endemic in the U.S. have received relatively little attention in a field setting. However, antibody prevalence to an orthopoxvirus (presumably raccoonpox virus) in raccoons of nearly 24% has been reported with positive hemagglutination inhibition antibody titers ranging from 1:80 to 1:2560 [19]. Because it appears that at least one naturally occurring orthopoxvirus can influence successful oral vaccination or rabies VNA titers following V-RG vaccinations, large-scale serosurveys are needed to determine the extent and prevalence of naturally occurring orthopoxviruses in the U.S and other areas where vaccinia-vectored wildlife vaccines are utilized for oral vaccination. This may require novel assay development to produce a test which can differentiate between vaccinia and other orthopoxviruses. Thus, an approach similar to published methods for West Nile virus and general flaviviruses [24] may be warranted (e.g., a species independent bELISA using multiple monoclonal antibodies). If evidence of exposure to orthopoxviruses is found at high natural prevalences, different vaccination strategies may be warranted in these areas for enhanced ORV success. These results may help to explain the relatively low vaccination rates of raccoons in some areas [1] if these endemic orthopoxviruses are found there at high prevalence. However, vaccination rates of raccoons are undoubtedly influenced by many other factors such as bait delivery, bait density, and bait uptake.

## Methods

### Animal husbandry and handling

Twenty adult raccoons (mass range: 4.2 – 9.0 kg at capture) were live-trapped in Larimer County, Colorado. Following capture, raccoons were de-wormed, dusted for ectoparasites, bled, and ear-tagged for individual identification. The subject animals were bled on a second occasion prior to experimentation and all were given a minimum of a two week quarantine period prior to the initiation of the experiment. Raccoons were individually housed in 10 × 10 × 8' animal pens. Food and water were replenished daily and general health checks were conducted each day. Animal procedures were approved by the National Wildlife Research Center's Animal Care and Use Committee.

Each time raccoons were handled, they were anesthetized with an intramuscular injection of a 5:1 mixture of ketamine/xylazine. For vaccination purposes, raccoons were only lightly anesthetized to allow for swallow reflexes. On day 0 of the experiment, 10 randomly chosen raccoons were administered 10^8 ^pfu of a raccoonpox virus-vectored plague vaccine (RCN-F1; [25]) *per os *to stimulate raccoonpox virus antibody production. To accomplish this, the vaccine was slowly administered into the oral cavity of the treatment animals with a 1 ml syringe. Following administration of the vaccine, the mouths of the animals were held closed for approximately ten seconds. On day 30 of the experiment, all raccoons were bled via the jugular vein. On day 33, all raccoons were vaccinated with 10^7 ^pfu of V-RG (titer range = 1 × 10^7 ^to 1.2 × 10^7^; see discussion). All raccoons were again bled on days 69 and 97. On day 118, all raccoons were vaccinated with a second dose of V-RG (10^8 ^pfu; titer range = 1 × 10^8 ^to 1.5 × 10^8^). All raccoons were bled again on days 139, 169, 200, 227, and 242 (Table 1). Blood samples were centrifuged to collect serum samples and these samples were stored at -70°C in cryovials prior to laboratory analyses. To save resources, serological assays were not conducted for some of the sampling time points.

**Table 1:** Experimental timeline for raccoon vaccination (RCN-F1 and Raboral V-RG^®^) and blood sample collection

Day	Activity
Pre-experiment	Raccoon capture, quarantine, and pre-experiment blood sample (blood sample 1)
-2	Second pre-experiment blood samples (blood sample 2)
0	10^8 ^pfu RCN-F1 administered to 10 raccoons at random
30	Blood sample 3
33	10^7 ^pfu Raboral V-RG^® ^administered to all raccoons
69	Blood sample 4
97	Blood sample 5
118	10^8 ^pfu Raboral V-RG^® ^administered to all raccoons
149^a^	Blood sample 6
179	Blood sample 7
210	Blood sample 8
237	Blood sample 9
252	Blood sample 10

^a^To save resources, serologic assays were not conducted on samples collected from all sampling occasions.

### Serological and plaque assays

Rabies VNA titers were assessed using rapid fluorescent focus inhibition tests (RFFIT) at a diagnostic laboratory. In addition, a microneutralization assay was used to assess raccoonpox virus antibody titers [25], although this assay appears to have some cross-reactivity with other orthopoxviruses (e.g., vaccinia). The raccoonpox virus serological assays were conducted at JEO's lab (University of Wisconsin). The detection threshold was 1:5 for the microneutralization test and 0.05 IU/ml for the RFFIT. Additionally, plaque assays were conducted to assess the titer of the V-RG vaccine using slight modifications of previously outlined methods [26]. In brief, the vaccine innoculum was 10-fold serially diluted in BA-1. One hundred microliters of each dilution was added in duplicate to Vero cell monolayers in 6-well plates. After 1 hour of incubation at 37°C, the cells were overlaid with 3 mL/well of 0.5% agarose in MEM medium supplemented with 5% fetal bovine serum, 350 mg/L sodium bicarbonate, 29.2 mg/L L-glutamine, 100 units/mL penicillin, 100 mg/L streptomycin, and 1 mg/L Fungizone, pH 7.6. After 48 hrs of additional incubation, a second 3 mL 0.5% agarose overlay containing 0.004% neutral red dye was added for plaque visualization. Plaques were counted on days 3 and 4 following infection of the Vero cells. The limit of detection of this assay was 10^1.7 ^PFU/mL.

### Post mortem examinations

On day 252, all raccoons were humanely euthanized and post-mortem examinations were conducted at the Colorado State University Veterinary Diagnostic Laboratory. Although these necropsies were performed for other purposes, some of the findings are mentioned in this paper for post hoc safety evaluations of the two vaccines administered to raccoons.

## Abbreviations

V-RG: Raboral V-RG^®^; VNA: virus neutralizing antibodies; ORV: oral rabies vaccination; DPI: day post inoculation; GMT: geometric mean titer

## Authors' contributions

JJR designed the research, performed the research, analyzed data, and wrote the paper. RGM provided conceptual efforts and designed research. DS analyzed data and wrote the paper. KAM performed the research. JEO performed the research and analyzed data.
